# Assessing *Jatropha curcas* pollen viability: a comparative assessment of transgenic and non-transgenic pollen under various environmental conditions using rapid staining technique

**DOI:** 10.3389/fpls.2025.1543947

**Published:** 2025-04-29

**Authors:** Sampath Kasthurirengan, Yan Hong, Srinivasan Ramachandran

**Affiliations:** ^1^ Temasek Lifesciences Laboratory, National University of Singapore, Singapore, Singapore; ^2^ JOil (S) Pte Ltd., National University of Singapore, Singapore, Singapore; ^3^ School of Biological Sciences, Nanyang Technological University, Singapore, Singapore

**Keywords:** pollen viability, FDA+PI combination, Jatropha pollen, X8#34 transgenic, non-transgenic

## Abstract

**Introduction:**

Pollen plays a critical role in transgene flow between non-transgenic plants, influencing gene dispersal and environmental risk considerations. Jatropha (*Jatropha curcas*) is a promising biofuel crop, offers an opportunity to study pollen biology, particularly in transgenic lines. Understanding pollen viability under different environmental conditions is essential for assessing potential risks associated with transgenic Jatropha cultivation.

**Methods:**

Pollen viability of X8#34 transgenic and non-transgenic Jatropha was assessed using various staining techniques. An optimized double-staining technique with Fluorescein Diacetate (FDA) and Propidium Iodide (PI) was developed, effectively differentiated viable pollen (green fluorescence) from non-viable (red fluorescence). The effects of temperatures (18°C, 28°C, 30°C 35°C, 40°C and 45°C) and UV-B irradiation (3 to 15 W/m2) on pollen viability ware examined. Additionally, viability was assessed under field-relevant conditions, including sunny and cloudy/shady atmospheric environments.

**Results:**

A significant reduction in pollen viability was observed under extreme temperature and UV-B stress. Among different temperatures tested, high temperatures (35°C, 40°C and 45°C) led to a significant decline in pollen viability, with notable differences emerging from 15 min of incubation. Additionally, exposure to high-intensity UV-B irradiation (12 W/m2 and 15 W/m2) significantly reduced the pollen viability. Under a field relevant sunny condition, viability dropped to 19% in transgenic and 16% in non-transgenic after 45 min incubation and complete loss was recorded in 90 min in both genotypes. In cloudy/shady conditions, over 97% of pollen lost viability in 240 min incubation. Statistical analysis confirmed no significant difference is pollen viability between X8#34 and non-transgenic Jatropha across all tested conditions.

**Discussion:**

This study provides the first comprehensive assessment of pollen viability in transgenic and non-transgenic Jatropha. The findings highlight the significant influence of environmental factors, particularly temperature and UV-B exposure, on pollen longevity. The optimized double staining technique (FDA + PI) provides a reliable method for assessing pollen viability and may be useful in environmental risk evaluations of transgenic Jatropha. Given the rapid decline in pollen viability under field-relevant conditions, the likelihood of transgene flow via pollen appears limited.

## 1 Introduction

Pollen is an important vector for gene flow, particularly for out crossing and essential for seed production. Cross-pollination by genetically modified (GM) plants through wind or foraging insects creates environment concern because of the possibility of transmitting transgene to their non-transgenic and counterparts and genetically compatible weeds. Risk assessment regarding transgene flow is an important issue to address for GM field trial and an important factor for commercial release. Pollen management is an important concern to limit transgene flow between transgenic and non-transgenic plants, and effective pollen management depends on good understanding of pollen biology especially its viability under natural conditions. Pollen viability is influenced by three main factors: (i) internal factors, such as metabolism; (ii) morphological factors; and (iii) environmental factors, such as temperature, relative humidity, and ultraviolet radiations in sunlight (Dowding, 1987; Dafni and Firmage, 2000; Luna et al., 2001). The sensitivity to high temperature at various stages of pollen development varies, which can result in pollen abortion and failure in seed setting (Rieu et al., 2017). In addition, UV-B is highly harmful and can induce various detrimental effects (Zedek et al., 2021). Plants, which require light for photosynthesis, are invariably exposed to UV-B radiation (Llorens et al., 2015). Consequently, this exposure can limit the plants’ growth and reproductive development, causing various adverse effects (Yadav et al., 2020; Piccini et al., 2021). While most studies have focused on vegetative development, it is important to note that plant reproductive structures, such as pollen grains, are particularly vulnerable to UV-B radiation and require more attention (Demchik and Day, 1996; Feng et al., 2000; Wang et al., 2010; Llorens et al, 2015). Understanding the duration of pollen viability is crucial for developing strategies to manage gene flow. Pollen viability is one of the crucial factors in GM plant risk assessment. Pollen viability is generally defined as pollen germination on the stigma, forming a pollen tube followed by compatible pollination. Assessment of pollen viability based on germinating capacity on the stigma is cumbersome, time consuming, and not always feasible. Staining with dyes and *in-vitro* germination of pollen on suitable conditions are becoming generally acceptable alternatives. Staining with dyes to assess pollen viability is based on enzymatic activity and plasma membrane integrity (Heslop-Harrison and Heslop-Harrison, 1970; Shivanna and Johri, 1985; Pacini et al., 1997).

The selection of pollen viability test depends on the crop or species (Dafni and Firmage, 2000; Dafni et al., 2005). Rodriguez-Riano and Dafni (2000) recommended the use of heat-killed pollen as a control to check the potential of the dye for testing pollen viability. Nuclear and vital dyes [Alexander’s procedure; acetocarmine; aniline blue; fluorescein diacetate (FDA); 2,3,5-triphenyl tetrazolium chloride (TTC)/2,5-diphenyl mono tetrazolium bromide (MTT); and X-Gal], which detect the presence of cytoplasm or enzymes intact, respectively, were used to determine the pollen viability (Rodriguez-Riano and Dafni, 2000; Nepi et al., 2005).

Pollen viability also assessed by *in-vitro* pollen germination needs the addition of certain mineral substrates such as calcium nitrate to the germination media in many species (Steer and Steer, 1989). Many pollen grains can germinate in water or in aqueous solutions of sucrose with no additives. However, the pollen of some species needs special substrates for germination. Germination of pollens in various concentrations of sucrose (usually between 0% and 50%) is often used to evaluate the osmotic relations of the pollen grains and to disclose the optimal concentration for germinability tests for each species (Dafni et al., 2005). The composition of the germination medium can dramatically affect pollen metabolism (Taylor and Hepler, 1997). Pollen viability in the family Euphorbiaceae has been studied in several genera (Pacini et al., 1997; Chang-Wei et al., 2007; Lyra et al., 2011; de Jesus Vieira et al., 2012; 2015; Abdelgadir et al., 2012; Cuchiara et al., 2015; Amkul et al., 2016). From our preliminary study of in *in-vitro* germination study of Jatropha pollen, during incubation in media pollen wall disintegrates from the pollen, and it was difficult to distinguish germinated and non-germinated pollens. Moreover, it was a time-consuming process. This prompted us to identify a rapid and robust method for routine assessment of access the pollen viability of *Jatropha curcas*. Jatropha is a monoecious shrub-producing unisexual flower, but some studies reported that Jatropha bears hermaphrodite flowers occasionally (Dehgan and Webster, 1978). Cymose type of inflorescence with yellowish-green flowers is arranged in terminal cymes. Normally, Jatropha male flowers start opening from the first or second day of the inflorescence life (13–19 days), while female flowers open later, with 60% of them opening from the third to fifth day (Changi-Wei et al., 2007; Achten et al., 2008; Divakara et al., 2010), and this flowering cycle continues in tropical climate conditions, while twice per year in the arid and semi-arid region. Jatropha is not only a self-pollinating species but also relies on insect-mediated pollination (Wang and Ding, 2012). Our observations indicate that cross-pollination occurs through pollen transporters, including ants and short-foraging insects, which are attracted to Jatropha flowers by nectar availability (Raju and Ezradanam, 2002; Banjo et al., 2006; Rianti et al., 2010). These insects play a significant role in pollen transfer over short distances, complementing wind-mediated dispersal (Sampath et al., 2025). During transfer through insects and wind dispersal, pollen viability may be influenced by multiple factors, including exposure to temperatures, ultraviolet radiation, and the time elapsed before reaching the stigma. These environmental factors may lead to pollen desiccation, and reduction in germination potential, ultimately impacting the fertilization process (Franchi et al., 2014). However, limited studies on pollination mechanisms within the Jatropha genus, and no extensive studies on staining methods to assess pollen viability in transgenic and non-transgenic *Jatropha curcas*. The transgenic high oleic acid content, marker-free Jatropha event X8#34 (Qu et al., 2012), has been subjected to an open field trial in Singapore to assess its environmental risk assessment, with a focus on pollen biology. In this study, an optimized rapid double dye staining technique (FDA+PI) was used to evaluate Jatropha pollen viability—different temperatures and different sunshine conditions (sunny and cloudy/shady) on Jatropha pollen viability. Finally, ultraviolet-B (UV-B) radiation at field-relevant dosages was used to treat pollen to assess its impact on Jatropha pollen viability.

## 2 Materials and methods

Pollens were harvested from X8#34 transgenic and non-transgenic Jatropha plants under the field trial at Semakau Island (1°12’34”N, 103°46’46”E), Singapore, between years 2015 and 2017 (in several quarters). Inflorescences were harvested in the morning (07:00–08:00h) with half-opened flower buds to avoid desiccation, and withering of flower bud inflorescences were immediately placed onto the container filled with distilled water for pollen extraction.

### 2.1 Tests for pollen viability by staining

A wide range of staining methods was evaluated to differentiate fresh pollen and dead pollen (incubated at 80°C for 2h in a glass Petri dish) from transgenic and non-transgenic plants. Stains preparation and methodologies followed the respective references in **Table 1**.

**Table 1:** Summary of different staining methods adapted for pollen viability assessment.

Stains	Viability differentiation	References
Aniline blue	Viable pollen turns to blue.	Khatun and Flowers (1995).
2,3,5-triphenyl tetrazolium chloride (TTC) or 2,5-diphenyl mono tetrazolium bromide (MTT)	Viable pollen turns to deep pink.	Khatun and Flowers (1995); Rodriguez-Riano and Dafni (2000).
Lugol solution (potassium iodide - KI)	Viable pollen turns to black.	Sasikala et al. (2009).
Acetocarmine	Viable pollen turns to red.	Lyra et al. (2011).
Fluoro chromatic reaction (FCR/FDA)	Viable pollen in green fluorescent.	Abdelgadir et al. (2012).
FDA + propidium Iodide (PI)	Viable pollen in green fluorescent; non-viable in red in color.	Duplakova et al. (2016); Cetinbas-Genc et al. (2022)

Pollens were gently dusted by tapping with a brush onto the glass slide containing a drop of stain. Then, they were gently agitated with a needle to ensure that the proper mixing of pollen with stain solution and to avoid air bubbles in the solution.

The preparation was kept under dark at room temperature to facilitate the reaction between pollen and dye, especially for FCR/FDA and FDA+PI. Pollens were viewed under microscope (Zeiss Imager.M2) with appropriate filters for FCR/FDA and FDA+PI. Viable and non-viable pollens were counted in each field of view for a total count of 100 pollens. Staining percentage was determined by dividing the number of stained pollens by the total number of pollens per field of view and expressed as a percentage.

### 2.2 Effect of different parameters on pollen viability

To test the effect of temperature on viability, pollens were dispensed onto the glass slide and incubated at different time points (0, 15, 30, 45, and 60 min) at 18°C, 28°C, 30°C, 35°C, 40°C, and 45°C in incubator with 55% relative humidity and without light before viability test.

Effect of ultraviolet radiation was performed by exposing pollen to UV-B light source for 10 min (13 W UV-B lamps, Exo-Terra) at 25 ± 2°C with 80% relative humidity before staining. The accumulated UV-B doses are 3, 6, 9, 12, and 15 W/m^2^ as measured by a UV-B–specific sensor (Tenmars, Taiwan). These dosages were within what plants receive under natural conditions.

Pollen viability under natural conditions was tested by exposing pollens to an open environment during non-rainy days. Pollen viability was assessed for 0, 15, 30, 45, 60, and 90 min under sunny conditions with 30°C–33°C temperature and 300–700 solar radiation W/m^2^ and 0, 15, 30, 45, 60, 90, 120, 150, 180, 240, and 300 min under cloudy/shady conditions with 26°C–28°C temperature and solar radiation of 10–200 W/m^2^.

### 2.3 Statistical analysis

Statistical analyses were carried out using GraphPad Prism software package, version 10.3.1. Each experiment was conducted at a randomized complete block design (25 plants were selected to harvest pollens) with replicates. The vertical error bars on data points represent the standard error and “•” denotes replicate values. The mean differences were carried out using analysis of variance (ANOVA) and Tukey’s HSD test, and significance was determined at the *P* < 0.05.

## 3 Results

### 3.1 Comparison of pollen viability by different staining methods

Jatropha pollen viability was assessed using different staining techniques, and the results are summarized in **Table 2**. Both X8#34 transgenic and non-transgenic pollen were effectively stained with aniline blue, acetocarmine, and potassium iodide (KI). However, these stains did not provide a clear distinction between viable (fresh) and non-viable (dead) pollen, as the color contrast was not sufficiently distinct. The stains, TTC/MTT, successfully penetrated the pollen grains. However, their effectiveness in viability assessment was limited due to the rapid fading of the stain, making visual differentiation unreliable. In contrast, the FDA dye provided a more effective means of viability assessment. Viable pollen exhibited a bright green fluorescence under excitation at 488 nm, whereas non-viable pollen remained unstained and was not visible. To enhance the accuracy of differentiation, a double-staining approach using FDA in combination with PI was employed. This FDA+PI double-staining method successfully distinguished viable and non-viable pollen, with viable pollen fluorescing green and non-viable pollens emitting a distinct red fluorescence when observed under excitation wavelengths of 488 nm and 649 nm, respectively (**Supplementary Figure S1**).

**Table 2:** Summary of different staining techniques in Jatropha pollen viability assessment of the X8#34 transgenic line and non-transgenic reference.

Stains	Differentiation of pollen		Results	Jatropha pollen staining techniques
	Viable	Dead		
Aniline blue	+	+	Unclear to differentiate between dead and viable pollen.	Abdelgadir et al. (2012).
TTC/MTT	+	–	Clearly differentiate into dead and viable pollen but stain fades quickly.	Abdelgadir et al. (2012); Kaur et al. (2011); Chang-Wei et al. (2007).
Lugol solution	+	+	Unclear to differentiate dead and viable pollen.	Abdelgadir et al. (2012); Sasikala et al. (2009).
Acetocarmine	+	+	Unclear to differentiate between dead and viable pollen.	Amkul et al. (2016); Tiwari et al. (2014); Lyra et al. (2011) in other Jatropha species.
FCR	+	–	Clearly stains viable pollen but dead pollen cannot be countable.	Abdelgadir et al. (2012).
FDA+PI	+	–	Able to differentiate both viable and dead pollen in fluorescent green and red in color, respectively.	In this study.

The FDA+PI double-staining protocol proved to be a reliable and robust technique for evaluating pollen viability in *Jatropha curcas*. Therefore, we considered that FDI + PI is an appropriate and robust technique for assessing changes in pollen viability due to environmental factors.

### 3.2 Effect of temperature on pollen viability

Pollen viability was evaluated across a range of temperatures that simulate field conditions. Freshly harvested X8#34 transgenic Jatropha and non-transgenic pollen were immediately distributed onto the glass slides and incubated at different temperature regime with different time period (0, 15, 30, 45, and 60 min) with 55% ± 2% relative humidity in the absence of light. Following incubation, treated pollens were stained with FDA+PI and incubated in the dark for 5 min to facilitate the reaction.

**Figure 1:**
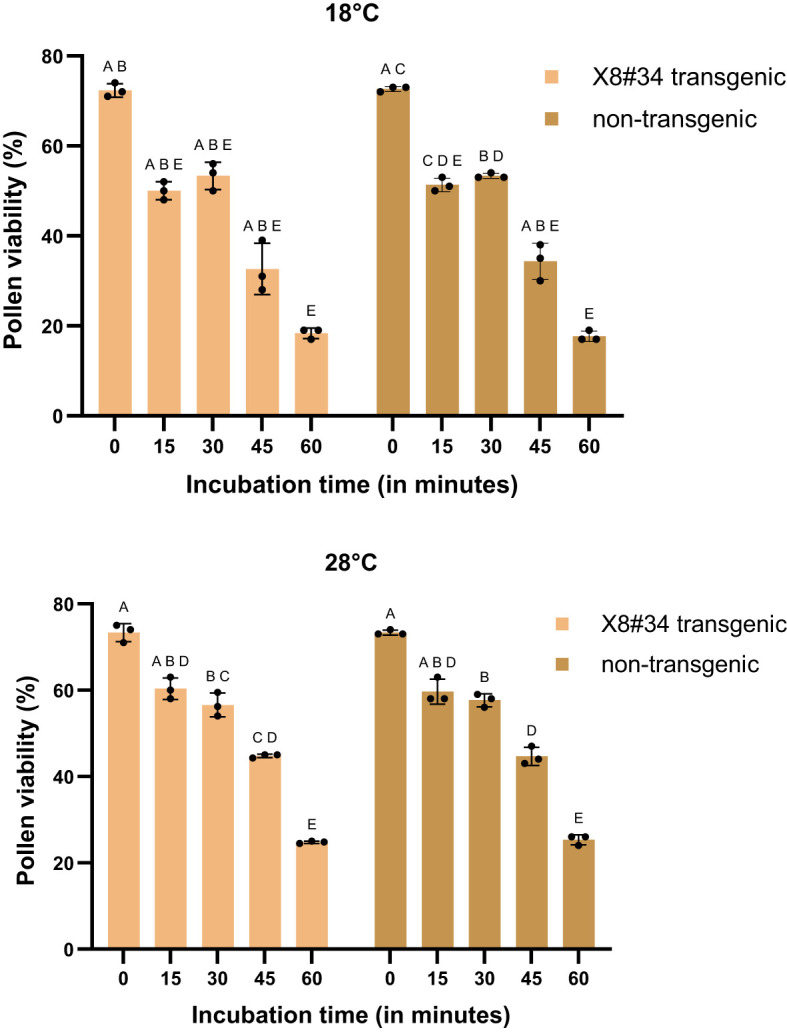
Effect of temperature (18°C and 28°C) on pollen viability in two genotypes (X8#34 transgenic and non-transgenic Jatropha pollen). Data represent means ± SE. Pollen from both genotypes was incubated for varying durations (0 to 60 min) before staining with FDA+PI. The letters above the bar represent statistical grouping based on a comparison means using Tukey’s HSD test. Bars sharing the same letter indicate no significant difference (P>0.05), whereas bars with different letters denote statistically significant differences in pollen viability between the two genotypes. P value for genotype X temperature interaction at 18°C and 28°C is P=0.6147 and P=0.5496 respectively.

Pollen viability varied depending on temperature and incubation duration. At 28°C, approximately 60%–70% of pollen remained viable after 15 and 30 min in both X8#34 transgenic and non-transgenic Jatropha (**Figure 1**). After 60 min incubation at 28°C, viability declined to 25% pollen in both genotypes: X8#34 transgenic and non-transgenic Jatropha. While no significant differences were observed between transgenic and non-transgenic pollen, viability percentages differed significantly compared to the control (0 min) at later time points (P** at 30 min; P** at 45 min; P*** at 60 min*). The difference observed at the 60-min incubation time is statistically significant, with a *p*-value likely between 0.001 and 0.05.

**Figure 2:**
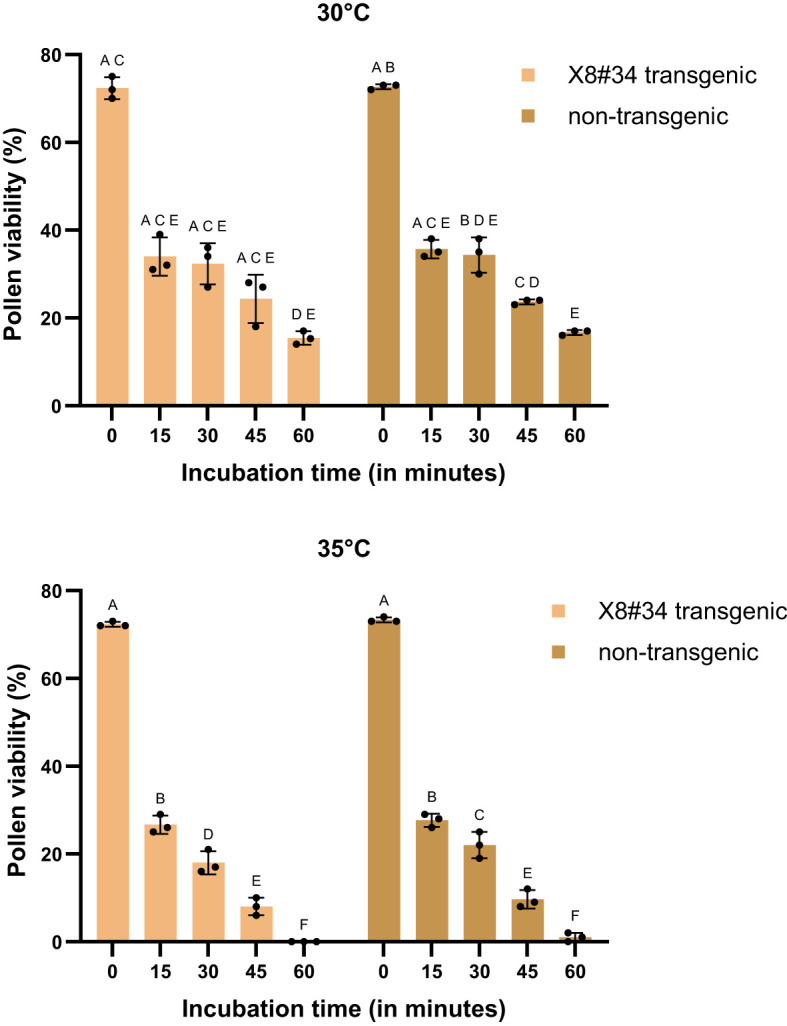
Effect of temperature (30°C and 35°C) on pollen viability in two genotypes (X8#34 transgenic and non-transgenic Jatropha pollen). Data represent means ± SE. Pollen from both genotypes was incubated for varying durations (0 to 60 min) before staining with FDA+PI. The letters above the bar represent statistical grouping based on a comparison means using Tukey’s HSD test. Bars sharing the same letter indicate no significant difference (P>0.05), whereas bars with different letters denote statistically significant differences in pollen viability between the two genotypes. P value for genotype X temperature interaction at 30°C and 35°C is P=0.5701 and P=0.041 (P*) respectively.

**Figure 3:**
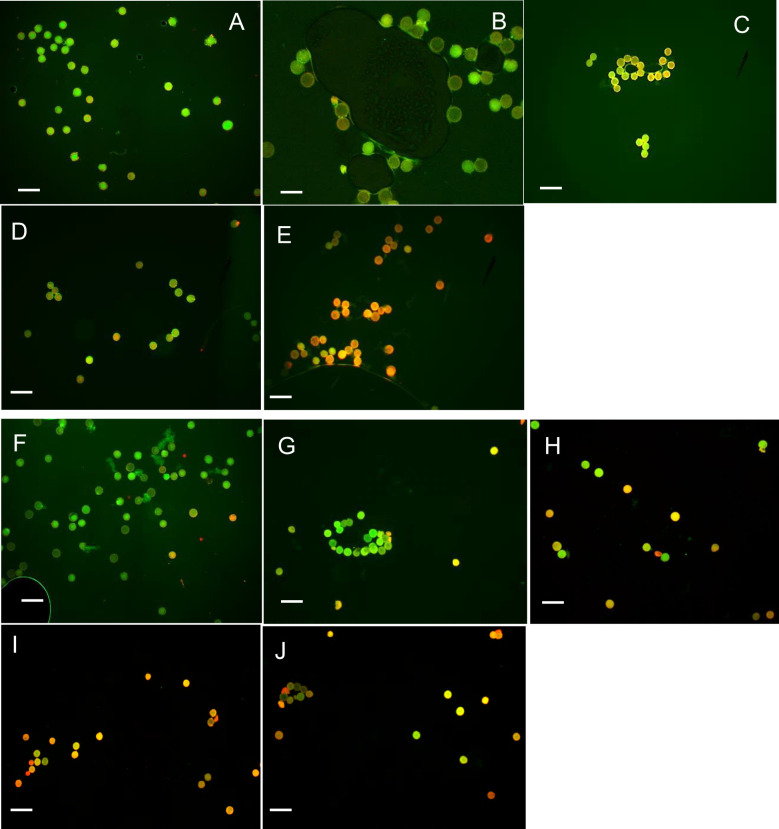
Effect of temperature (30°C) on pollen viability of X8#34 transgenic Jatropha and non-transgenic Jatropha. **(A)** X8#34 transgenic pollen - control without temperature treatment. **(B–E)** X83#4 transgenic pollen treated at 30°C at various time points (15, 30, 45 & 60 min). **(F)** Non-transgenic pollen - control without temperature treatment. **(G–J)** Non-transgenic pollen treated at 30°C at various time points (15, 30, 45 & 60 min), (bar = 100 µm).

Under temperature treatments, decline in pollen viability was observed at temperature of 30°C and above, with a significant reduction begins at 15 min (**Figure 2**). At 60 min, viability was significantly lower (P***, *P* < 0.0001*). At 35°C treatment, less than 10% remained viable beyond 45 min incubation (**Figures 2**, **3**). The difference in percentages between 30 min and 45 min was statistically significant (P**, *P* = 0.0002*, and *P*, *P* = 0.0308*, respectively).

**Figure 4:**
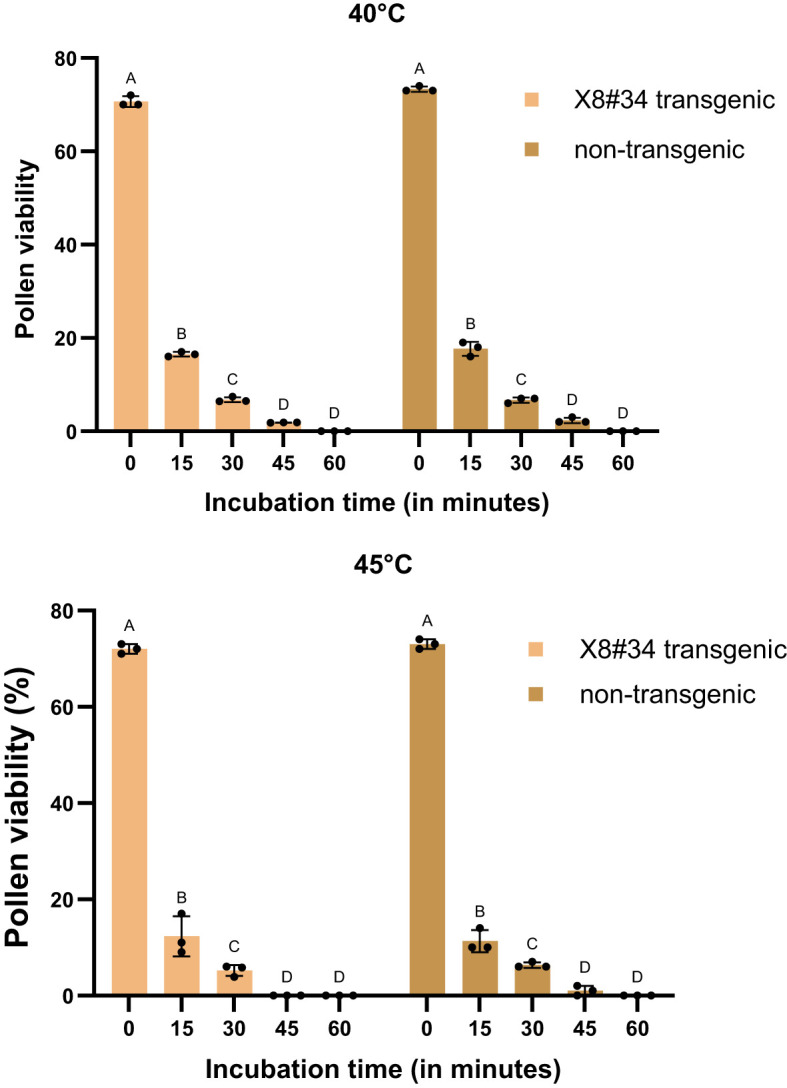
Effect of temperature (40°C and 45°C) on pollen viability in two genotypes (X8#34 transgenic and non-transgenic Jatropha pollen). Data represent means ± SE. Pollen from both genotypes was incubated for varying durations (0 to 60 min) before staining with FDA+PI. The letters above the bar represent statistical grouping based on a comparison means using Tukey’s HSD test. Bars sharing the same letter indicate no significant difference (P>0.05), whereas bars with different letters denote statistically significant differences in pollen viability between the two genotypes. P value for genotype X temperature interaction at 40°C and 45°C is P=0.0721 and P=0.4792 respectively.

**Figure 5:**
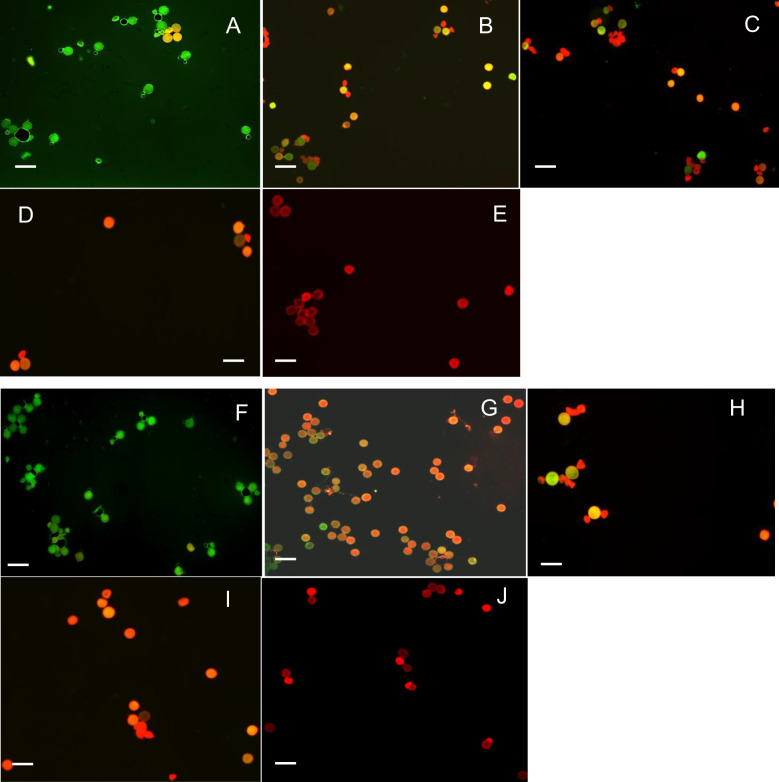
Effect of temperature (45°C) on pollen viability on X8#34 transgenic Jatropha and non-transgenic Jatropha. (A) X8#34 transgenic pollen - control without temperature treatment. **(B–E)** X8#34 transgenic pollen treated at 45°C at various time points (15, 30, 45 & 60 min). **(F)** Non-transgenic pollen - control without temperature treatment. **(G–J)** Non-transgenic pollen treated at 45°C at various time points (15, 30, 45 & 60 min), (bar = 100 µm).

When pollens were treated under extreme temperature conditions (40°C and 45°C), pollen viability declined sharply. At 45°C, X8#34 transgenic became 100% non-viable within 45 min (**Figures 4**, **5**), whereas a small fraction (~2%) of non-transgenic pollen remained viable. Across all temperature treatments, no significant differences were detected between the genotypes tested: X8#34 transgenic and non-transgenic Jatropha pollen viability. Control samples for both exhibited similar level of viability percentage throughout the study.

**Figure 6:**
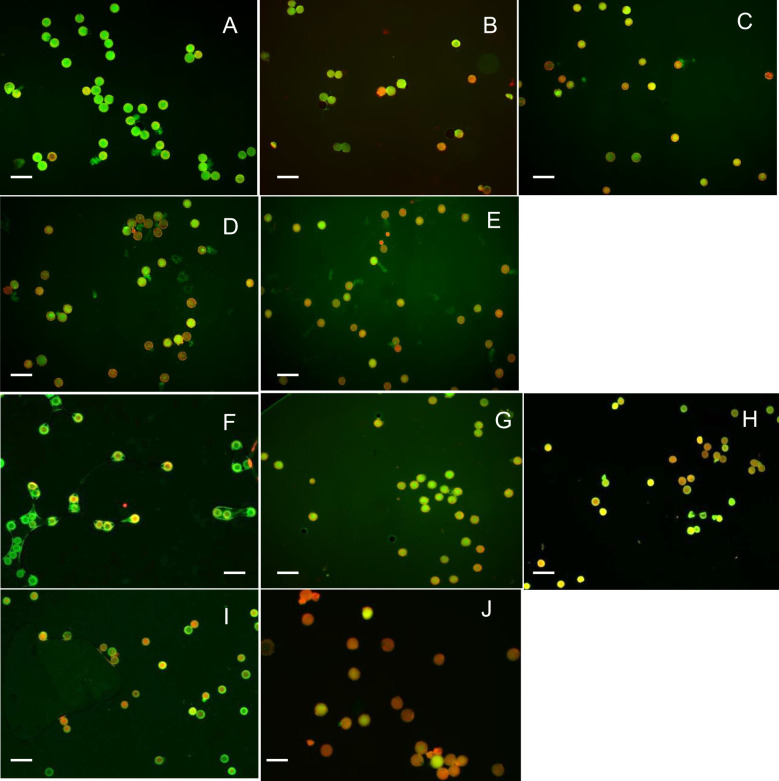
Effect of temperature (18°C) on pollen viability of X8#34 transgenic Jatropha and non-transgenic Jatropha. **(A)** X8#34 transgenic pollen - control without temperature treatment. **(B–E)** X8#34 transgenic treated at 18°C at various time points (15, 30, 45 & 60 min). **(F)** Non-transgenic pollen - control without temperature treatment. **(G–J)** Non-transgenic pollen treated at 18°C at various time points (15, 30, 45 & 60 min), (bar = 100 µm).

Environmental factors, particularly temperature, strongly influenced, pollen viability during the flowering period of *Jatropha curcas*. At 18°C and 30°C, approximately 50%–60% of pollen remained viable for short incubation periods (15 and 30 min) (**Figures 1**, **2**, **6**, and **3**). However, at 45°C, viability dropped below 20% (**Figures 4**, **5**). ANOVA showed that genotype × temperature (G × T) interaction on pollen viability was also observed. The ANOVA results suggested that significant interaction (P****) between genotype × temperature. While genotype had a less significant effect on pollen viability, its influence was considerably weaker than that of temperature (**Tables 3**, **4**), indicating that temperature is the prime factor affecting pollen viability.

**Table 3:** Analysis of variance (ANOVA) for the interactions of Genotype (G) × Temperature (T) on Jatropha pollen viability at different incubation times (0, 15, 30, 45, and 60 min).

ANOVA	Sum Sq.	Df	Mean Sq.	F (DFn	DFd)	*P*-value
Temp × Genotypes	9792	45	217.6	F (45	100) = 53.36	*P* < 0.0001
Temp	29481	5	5896	F (2.769	55.38) = 1446	*P* < 0.0001
Genotypes	83223	9	9247	F (9	20) = 1344	*P* < 0.0001
Residual	407.8	100	4.078			

**Table 4:** Tukey’s multiple comparisons tests for two genotypes (X8#35 transgenic and non-transgenic Jatropha) on pollen viability at different temperature treatment and time points (min).

Tukey's multiple comparisons test	Mean Diff.	95.00% CI of diff.	Below threshold	Summary	*P Value*
18°C					
non-transgenic (0) vs. transgenic (0)	0.3333	-6.869 to 7.536	No	ns	>0.9999
non-transgenic (15) vs. transgenic (15)	1.333	-7.028 to 9.695	No	ns	0.984
non-transgenic (30) vs. transgenic (30)	0	-16.40 to 16.40	No	ns	>0.9999
non-transgenic (45) vs. transgenic (45)	1.667	-22.03 to 25.36	No	ns	>0.9999
non-transgenic (60) vs. transgenic (60)	-0.6667	-5.884 to 4.551	No	ns	0.9971
28°C					
non-transgenic (0) vs. transgenic (0)	0	-10.54 to 10.54	No	ns	>0.9999
non-transgenic (15) vs. transgenic (15)	-0.6667	-13.03 to 11.70	No	ns	>0.9999
non-transgenic (30) vs. transgenic (30)	1.083	-10.77 to 12.94	No	ns	0.9988
non-transgenic (45) vs. transgenic (45)	-0.1	-11.24 to 11.04	No	ns	>0.9999
non-transgenic (60) vs. transgenic (60)	0.5667	-5.522 to 6.655	No	ns	0.9858
30°C					
non-transgenic (0) vs. transgenic (0)	0.3333	-12.84 to 13.50	No	ns	>0.9999
non-transgenic (15) vs. transgenic (15)	1.667	-17.67 to 21.00	No	ns	0.9987
non-transgenic (30) vs. transgenic (30)	2	-18.14 to 22.14	No	ns	0.9995
non-transgenic (45) vs. transgenic (45)	-0.6667	-31.50 to 30.16	No	ns	>0.9999
non-transgenic (60) vs. transgenic (60)	1.233	-5.832 to 8.299	No	ns	0.8909
35°C					
non-transgenic (0) vs. transgenic (0)	1	-1.609 to 3.609	No	ns	0.5864
non-transgenic (15) vs. transgenic (15)	1	-7.680 to 9.680	No	ns	0.9978
non-transgenic (30) vs. transgenic (30)	4	-8.894 to 16.89	No	ns	0.751
non-transgenic (45) vs. transgenic (45)	1.667	-7.565 to 10.90	No	ns	0.9752
non-transgenic (60) vs. transgenic (60)	1	-4.713 to 6.713	No	ns	0.7526
40°C					
non-transgenic (0) vs. transgenic (0)	2.667	-2.397 to 7.731	No	ns	0.2425
non-transgenic (15) vs. transgenic (15)	1.167	-6.296 to 8.629	No	ns	0.9089
non-transgenic (30) vs. transgenic (30)	-0.1	-2.653 to 2.453	No	ns	>0.9999
non-transgenic (45) vs. transgenic (45)	0.4667	-2.771 to 3.704	No	ns	0.8642
non-transgenic (60) vs. transgenic (60)	0.3333	-2.965 to 3.632	No	ns	0.9616
45°C					
non-transgenic (0) vs. transgenic (0)	1	-3.519 to 5.519	No	ns	0.9317
non-transgenic (15) vs. transgenic (15)	-1	-18.84 to 16.84	No	ns	>0.9999
non-transgenic (30) vs. transgenic (30)	1.1	-3.988 to 6.188	No	ns	0.8471
non-transgenic (45) vs. transgenic (45)	1	-4.713 to 6.713	No	ns	0.7526
non-transgenic (60) vs. transgenic (60)	0				

ns, no significant.

Based on our observation, environmental factors—high temperature, influence pollen viability and contribute to reduction in fruit sets and yield in Jatropha. We have noted that high temperatures (>33°C) with low ground moisture level, which adversely affects the flower fertilization, causes immature fruit abortion, and in some cases, resulted in lack of endosperms (empty seeds), between 10% and 15% reduction in yield. Therefore, pollen viability is positively correlated with seed production, alongside of other factors, agronomic practices.

### 3.3 Effect of UV-B irradiation on pollen viability

**Figure 7:**
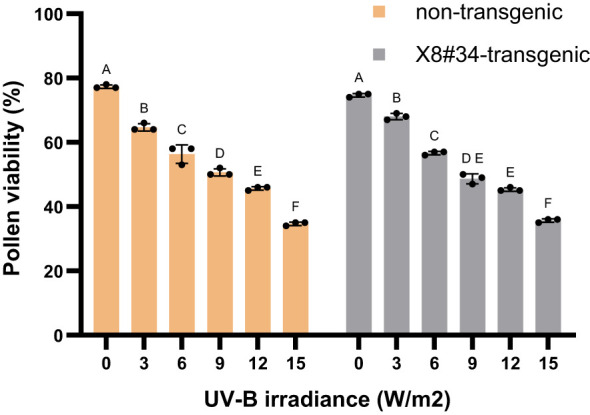
Effect of UV-B irradiation on pollen viability in X8#34 transgenic and non-transgenic Jatropha (means ± SE). Pollen was irradiated for 10 min at different accumulated doses of UV-B before staining with FDA+PI.. Data represent means ± SE. The letters above the bar represent statistical grouping based on a comparison means using Tukey’s HSD test. Bars sharing the same letter indicate no significant difference (P>0.05), whereas bars with different letters denote statistically significant differences in pollen viability between the two genotypes. P value is 0.9139, r=0.98.

UV-B radiation (280–320 nm) is normally present in the sunlight that reaches the earth’s surface, and it is therefore likely that mechanisms are present in plants to protect them against the damaging effects of UV-B. However, exposure to elevated UV-B levels negatively impact pollen viability, indicating that ultraviolet irradiation may have contributed to the reduction in pollen viability under direct sunlight. X8#34 transgenic and non-transgenic pollen were exposed to UV-B irradiation at room temperature (25 ± 2°C) for 10 min (**Figure 7**). At a lower UV-B irradiance of 3 W/m^2^, both X8#34 transgenic and non-transgenic pollen exhibited a higher percentage of viability. However, at 15 W/m^2^, pollen viability declined to 36.3% in X8#34 transgenic and 33% in non-transgenic Jatropha pollen. A dose-dependent increase in viability was observed, with significant inhibition occurring at higher UV-B doses of 12 and 15 W/m^2^ (**Figure 7**). Statistical analysis using ANOVA revealed no significant difference in pollen viability between in two genotypes: X8#34 transgenic and non-transgenic across all UV-B treatments (*P* = 0.9139, *P* > 0.05; *r* = 0.98). Additionally, UV-B dosages x genotype showed no significant effect on pollen viability (*P* = 0.1384). These suggests indicating that the observed decline in pollen viability is primarily due to variations in UV-B exposure rather than any genotypic differences (**Supplementary Table S1**).

### 3.4 Pollen viability under different atmospheric conditions

We evaluated viability of pollens from X8#34 transgenic and non-transgenic plants under sunny (direct sunlight) and cloudy/shady conditions in open field conditions.

**Figure 8:**
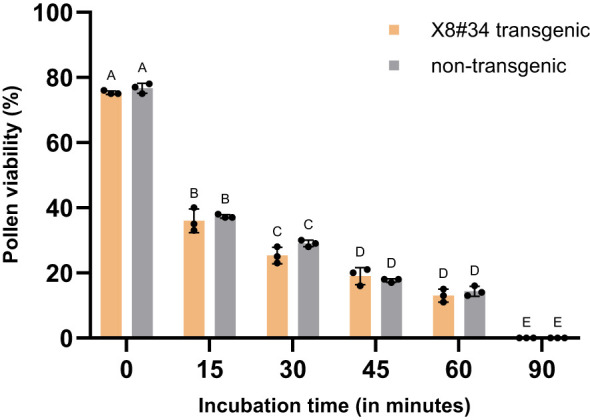
Viability of pollen from X8#34 transgenic and non-transgenic Jatropha under different atmospheric conditions (means ± SE). Pollen viable was evaluated under sunny conditions (direct sunlight) after exposure with different time points (in min) and evaluated by staining with FDA+PI. Data represent means ± SE. The letters above the bar represent statistical grouping based on a comparison means using Tukey’s HSD test. Bars sharing the same letter indicate no significant difference (P>0.05), while bars with different letters denote statistically significant differences in pollen viability between the two genotypes. P value is 0.048, r=0.99.

Under direct sunlight (30 ± 2°C with RH 40 ± 5%), pollen viability declined rapidly. Approximately 60% of both X8#34 transgenic and non-transgenic pollen lost viability within 15 min, and less than 20% remained viable after 30 min (**Figure 8**). By 90 min, pollen viability was completely lost in both genotypes: X8#34 transgenic and non-transgenic pollen. Statistical analysis indicated no significant difference in pollen viability between X8#34 transgenic and non-transgenic pollen (*P* > 0.05). ANOVA confirmed that the difference between the two genotypes was not statistically significant (*P* = 0.048; *r* = 0.99). Additionally, sunlight exposure × genotype had no significant impact on pollen viability (*P* = 0.3747). As the *P* value exceeded the significance threshold (*P* < 0.05), the observed variations are likely due to random variation rather than a genotype effects (**Supplementary Table S2**).

**Figure 9:**
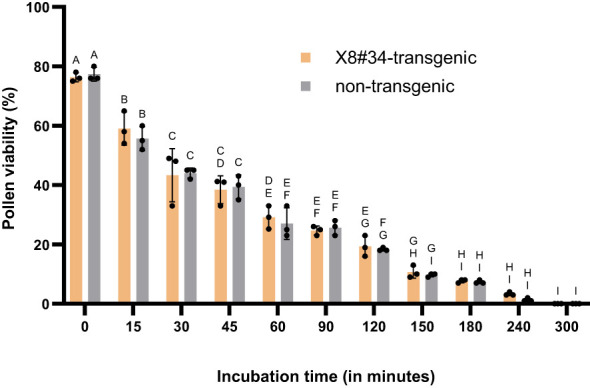
Viability of pollen from X8#34 transgenic and non-transgenic Jatropha under different atmospheric conditions (means ± SE). Pollen viable was evaluated under cloudy/shady conditions after exposure with different time points (in min) and evaluated by staining with FDA+PI. Data represent means ± SE. The letters above the bar represent statistical grouping based on a comparison means using Tukey’s HSD test. Bars sharing the same letter no significant difference (P>0.05), while bars with different letters represent statistically significant differences in pollen viability between the two genotypes. P value is 0.5811, r = 0.99.

In contrast, under cloudy/shady conditions (26 ± 2°C with RH 60 ± 2%), the decline in pollen viability was more gradual. After 15 min, approximately 60% of X8#34 of transgenic and 55% of non-transgenic pollens remained viable. However, viability declined to less than 10% after 180 min, and complete loss of viability after 240 min (**Figure 9**). A comparison of pollen viability between two genotypes (X8#34 transgenic and non-transgenic Jatropha) showed no significant difference (*P* = 0.5811; *r* = 0.99). However, when viability was compared between the control (0 min) and other incubation time points, a significant difference was observed at *P* < *0.05* level. Additionally, the interaction between cloudy/shady conditions and genotype had no significant effect on pollen viability (*P* = 0.9442), indicating that environmental factors rather than genotype primarily influenced pollen viability over time (**Supplementary Table S3**). Our results demonstrate that both X8#34 transgenic and non-transgenic Jatropha pollens rapidly lose viability under field conditions. Complete loss of viability occurs within 90 min under sunlight and within 240 min in cloudy/shady environmental conditions.

These results suggest that pollen viability in X8#34 transgenic and non-transgenic Jatropha is primarily influenced by external environmental factors rather than genetic background. Consequently, pollen viability loss in natural field conditions occurs similarly in both genotypes, reducing potential gene flow risks associated with transgenic Jatropha cultivation.

## 4 Discussion

Assessing pollen viability and longevity in transgenic pollen is a critical component of risk assessment (Wang and Ge, 2006; Wang et al., 2004). Developing a reliable method for testing pollen viability is crucial for advancing the study of pollination biology. While certain staining techniques can selectively stain only viable pollen in some plant species (Khatun and Flowers, 1995; Rodriguez-Riano and Dafni, 2000) their effectiveness can vary. In this study, we evaluated different staining methods including aniline blue, acetocarmine, TTC/MTT, KI, FDA, and FDA+PI. Among these, the FDA+PI combination clearly distinguished between viable and non-viable Jatropha pollen. Our observation aligns with Duplakova et al. (2016), successfully employed FDA+PI to screen male gametophytes in Arabidopsis. FDA+PI is considered an indirect method for assessing pollen viability as it evaluates membrane integrity. Cetinbas-Genc et al. (2022) reported that the FDA + PI combination was found to effective in differentiating viable and non-viable Hazelnut pollens after UV-B radiation treatment. Likewise, Impe et al. (2020) integrated the FDA staining and impedance flow (IF) cytometry methods to assess wheat pollen viability, providing reasonable indications of the pollen ability to germinate and grow. Soto-Landeros et al. (2017) reported that exine structures, as a well-developed exine, can protect pollen from desiccation and UV radiation. The cross-linked endexine observed in all Jatropha species may contribute to maintaining viability by reducing moisture loss. In the conditions, FDA based staining method could be effective differentiate the viable and non-viable pollen based on membrane integrity.

While FDA alone effectively detected viable Jatropha pollen by fluorescent green, it was unable to differentiate non-viable pollen from the mass. This finding is consistent with earlier studies (Shivanna and Heslop-Harrison, 1981; Heslop-Harrison et al., 1984; Shivanna et al., 1991; Dafni, 1992; Kearns and Inouye, 1993; Pacini et al., 1997, Li et al., 2010) and corroborates observation by Abdelgadir et al. (2012). Similarly, Ge et al. (2011) reported that FDA staining method often fail to accurately differentiate viability levels in switch grass. Similarly, Impe et al. (2020) evaluated the viability of fresh wheat pollen using FDA staining method and found that approximately 80% of the pollen exhibited with green fluorescence. However, to assess non-viable and stored pollen, additional methods such as an *in-vitro* germination were employed. Moreover, FDA staining has been successfully utilized to be assess pollen viability in various plant species, including cherry (La Porta and Roselli, 1991), potato (Trognitz, 1991), and acacia (Sedgley and Harbard, 1993).

Our findings on TTC/MTT stains, which effectively differentiates viable and non-viable pollen, however, the stain fades quickly. In contrast, Chang-Wei et al. (2007); Kaur et al. (2011); Abdelgadir et al. (2012) reported that TTC differentiates fresh and dead Jatropha pollen. While our results confirmed MTT/TTC’s ability to differentiate viable from non-viable pollen, the rapid fading limits the practical application. Further, we have observed that aniline-blue and KI failed to differentiate the fresh and dead pollen, consistent with the findings of Abdelgadir et al. (2012). However, Sasikala et al. (2009) reported that KI produced better results for pollen fertility in ten *Jatropha* species and an interspecific hybrid (*J. curcas × J. integerrima*). They reported that fully stained pollens are fertile, nine species with 84% fertile pollen, the hybrids at 97%, and *J. tanjorensis* exhibiting only 0.16%, indicative of near sterility. We have observed that acetocarmine failed to differentiate fresh and dead pollen and also observed with overstaining of pollen, which appears black in color under the microscope, resembling dead pollen. Despite its limitations, Basha and Sujatha (2009) successfully deployed for *Jatropha curcas* and nine other Jatropha species, while. Lyra et al. (2011) applied it to assess pollen viability of *J. ribifolia* and *J. mollissima*. Arzani et al. (2000) reported that the use of acetocarmine for staining both immature and adult pollen grains in the Triticeae family is tedious and often yields unsatisfactory results. Similarly, Parfitt and Ganeshan (1989) observed that acetocarmine stained non-viable pollen of genus *Prunus*, making it less reliable for distinguishing viable from non-viable pollen compared to other staining techniques.

Structural adaptations in Jatropha species pollen are not only essential for wind-mediated dispersal but also a play a critical role in maintaining pollen viability. The presence of luminal bascules in a *Jatropha curcas* pollen may retain moisture better than other Jatropha species, potentially contributing to higher viability under specific environmental conditions. These factors are crucial considerations for the risk assessment of genetically modified Jatropha field trials and large-scale commercial release.

Based on the aforesaid facts, our study was to develop a robust staining technique that provides a clear and reliable differentiation between viable and non-viable pollen. This is particularly important for evaluating pollen viability under diverse environmental conditions, which is essential in large-scale breeding programs, reproductive biology studies, and mitigating potential risks associated with genetically modified Jatropha field trials. Our findings indicate that the FDA +PI double-staining methods are the most effective technique for accurately differentiate fresh, viable, and non-viable pollen.

Wang and Ding (2012) provided further insights into pollen viability in *Jatropha curcas* by examining the influence of flower type and seasonal variations. Their study emphasized that pollen viability fluctuates depending on environmental conditions and the reproductive stage of the plant. Environmental factors significantly influence the pollen viability during the flowering period of *Jatropha curcas*. Our findings show that the fresh pollen remains viable at temperatures between 18°C and 30°C for shorter incubation periods; however, viability declines sharply at higher temperature (40°C and 45°C). Similar temperature dependent pollen viability has been observed in other plants, such as sorghum and tall fescue (*Festuca arundinacea*) (Tuinstra and Wedel, 2000; Wang et al., 2004). Our results indicate that temperature has a negative impact on *Jatropha curcas* pollen viability, even in the absence of factors like UV-irradiation and desiccation*. S*tudies on the effect of temperature stress in canola have revealed that pollen staining is reliable than pollen germination method (Rao et al., 1992; Young et al., 2004). Singh et al. (2008) reported that 12 field-grown canola cultivars displayed higher percentage using staining techniques (61.3% to 89.7%) than *in-vitro* pollen germination on solid media (29%–48.2%).

In addition, to temperature, UV irradiation also plays a significant role in reducing pollen viability under direct sunlight. Wang et al. (2004) reported no significant impact at lower dosage of UV-B irradiation in tall fescue (*Festuca arundinacea*), but higher UV-B doses significantly reduced pollen viability, suggesting that ultraviolet irradiation contributes to decreased pollen longevity under direct sunlight. Furthermore, increased UV-B exposure, caused by stratospheric ozone depletion, could affect pollen-viability in certain geographic regions. Detailed studies on UV-B effects in Brassica have demonstrated a decline in reproductive success (Demchik and Day, 1996; Feldheim and Conner, 1996; Conner and Zangori, 1997). Similarly, Feng et al. (2000) observed inhibition and reduction in *in-vitro* pollen germination in most species across 19 taxa studied, with cumulative inhibition of tube growth under prolonged UV-B exposure. Our study also corroborated with the studies reported that UV-B induces decreased pollen viability, in crop species such as soybeans, olives, and *Silene* (Sailaja et al., 2005; Koubouris et al., 2015; Del Valle et al., 2020; Tushabe et al., 2023).

Atmospheric conditions also drastically influence Jatropha pollen longevity. Under sunny atmospheric conditions, approximately 60% of pollen viability was lost within 15 min, with complete loss observed in 90 min for both transgenic and non-transgenic Jatropha pollen. In contrast, under cloudy atmospheric conditions, pollen viability declined by about 45%, with the complete loss occurring in 300 min. Comparatively, tall fescue (*Festuca arundinacea*) pollen can remain viable for nearly 3 days in open air environment (Pacini et al., 1997), while wheat pollen losses viability within 65–70 min and triticale within 110–120 min (Fritz and Lukaszewski, 1989). Maize pollen, however, exhibited no viability after 2h of atmospheric exposure (Luna et al., 2001).

Pollen viability is closely linked to seed yield, with environmental stresses, particularly high temperatures, significantly affecting seed production. Elevated temperatures can disrupt multiple reproductive stages, including flower development, pollen viability, fertilization, and seed filling, ultimately leading to reduced seed yield and quality. Among these factors, high temperatures have the most detrimental impact on seed production, primarily by impairing pollen function. A strong correlation between pollen viability, seed set, and grain yield has been reported in wheat (Yadav et al., 2020; Khan et al., 2022; Masthigowda et al., 2022; Mamrutha et al., 2020; Nasehzadeh and Ellis, 2017; Prasad et al., 2006; Saini et al., 1983; Saini and Aspinall, 1982). Pollen grains with high viability and high germination capacity are necessary for fertilization because non-viable pollen grains cannot form pollen tubes and cannot transfer genetic material to the embryo sac for fertilization (Shivanna et al., 1991), which directly affects the seed set and reduction in the yield.

Collectively, our studies underscore the evaluating the pollen viability under diverse environmental conditions to mitigate the risks associated with the genetically modified Jatropha field trials. Environmental factors such as temperatures, UV-B irradiation, and atmospheric conditions play pivotal roles in pollen viability, and their adverse effects on pollen can lead to significant reductions in seed yield.

There are no significant differences were observed in pollen viability and longevity between X8#34 transgenic and non-transgenic *Jatropha curcas*. This finding suggests that the physiological and reproductive characteristics of transgenic pollen closely resemble those of non-transgenic pollen. Consequently, insights derived from studying the viability and longevity of non-transgenic pollen can be reliably extrapolated to transgenic pollen, providing confidence in the comparability of their reproductive behaviors under similar environmental conditions.

## 5 Conclusion

Jatropha is not intended for human food or animal feed, which shifts the focus to its environmental impact —an area where pollination biology plays a pivotal role. In this study, we conducted a comprehensive comparison of pollen viability between the X8#34 transgenic Jatropha and its non-transgenic counterpart. Our findings demonstrate that there is no statistically significant difference in the viability of pollen between the X8#34 transgenic and non-transgenic Jatropha genotypes, suggesting that the genetic modification does not influence pollen performance.

To assess pollen viability, we employed an optimized and effective double-staining protocol using FDA + PI, which allowed clear differentiation between viable and non-viable pollen. This method enabled us to evaluate the impact of various environmental factors on Jatropha pollen viability. Under natural lighting conditions, pollen viability declined rapidly, with a lifespan of approximately 90 min under full sunlight and up to 240 min under cloudy conditions.

These findings provide valuable insights into the pollination biology of Jatropha and contribute to understanding the environmental dynamics of X8#34 transgenic and non-transgenic Jatropha pollen. This knowledge is essential for risk assessment and management in genetically modified crop trials, supporting informed in decision making for sustainable cultivation practices.

## Data Availability

The original contributions presented in the study are included in the article/**Supplementary Material**. Further inquiries can be directed to the corresponding authors.
