# Supplementary material for: Assessing Jatropha curcas pollen viability: a comparative assessment of transgenic and non-transgenic pollen under various environmental conditions using rapid staining technique
Source: Front Plant Sci. 2025 Apr 29;16:1543947. doi: 10.3389/fpls.2025.1543947 (PMC12069449; doi:10.3389/fpls.2025.1543947)

**Supplementary Materials:**

**Assessing *Jatropha curcas* pollen viability: A comparative assessment of transgenic and non-transgenic pollen under various environmental conditions using rapid staining technique.**

**Kasthurirengan Sampath ^1,2^, Hong Yan ^2,3*^ and Srinivasan Ramachandran^1,2*^**

^1^Temasek Lifesciences Laboratory, 1 Research Link, National University of Singapore, Singapore

^2^JOil (S) Pte Ltd, 1 Research Link, National University of Singapore, Singapore 117604.

^3^ School of Biological Sciences, Nanyang Technological University, Singapore 637551.

**Supplementary Table S1:**

Analysis of variance (ANOVA) for the interactions of Genotype X UV-B dosages on pollen viability.

| ANOVA table | SS | DF | MS | F (DFn | DFd) | *P value* |
| --- | --- | --- | --- | --- | --- | --- |
| UV-B dosages | 6502 | 5 | 1300 | F (1.125 | 2.250) = 1059 | P=0.0005 |
| Genotypes | 0.02778 | 1 | 0.02778 | F (1.000 | 2.000) = 0.01493 | P=0.9139 |
| UV-B x Genotypes | 35.14 | 5 | 7.028 | F (1.434 | 2.869) = 4.231 | P=0.1384 |
| Subject | 0.7222 | 2 | 0.3611 |  |  |  |
| Residual | 16.61 | 10 | 1.661 |  |  |  |

**Supplementary Table S2:**

Analysis of variance (ANOVA) for the interactions of Genotype X atmospheric conditions - sunny on pollen viability at different incubation times (0, 15, 30 , 45, 60 and 90 min).

| ANOVA table | SS | DF | MS | F (DFn | DFd) | *P value* |
| --- | --- | --- | --- | --- | --- | --- |
| Time points (sunny) | 20761 | 5 | 4152 | F (5 | 10) = 1104 | P<0.0001 |
| Genotypes | 10.03 | 1 | 10.03 | F (1 | 2) = 19.00 | P=0.0488 |
| Time points x Genotypes | 20.81 | 5 | 4.161 | F (5 | 10) = 1.202 | P=0.3747 |
| Subject | 0.7222 | 2 | 0.3611 |  |  |  |
| Residual | 34.61 | 10 | 3.461 |  |  |  |

**Supplementary Table S3:**

Analysis of variance (ANOVA) for the interactions of Genotype X atmospheric conditions – cloudy/shady on pollen viability at different incubation times (0, 15, 30 , 45, 60 and 90 min).

| ANOVA | SS | DF | MS | F (DFn | DFd) | *P value* |
| --- | --- | --- | --- | --- | --- | --- |
| Time points (cloudy/shady) | 35290 | 10 | 3529 | F (10 | 20) = 238.9 | P<0.0001 |
| Genotypes | 5.242 | 1 | 5.242 | F (1 | 2) = 0.4255 | P=0.5811 |
| Time points x Genotypes | 32.39 | 10 | 3.239 | F (10 | 20) = 0.3732 | P=0.9442 |
| Subject | 0.5758 | 2 | 0.2879 |  |  |  |
| Residual | 173.6 | 20 | 8.68 |  |  |  |

**Supplementary Figure S1:**


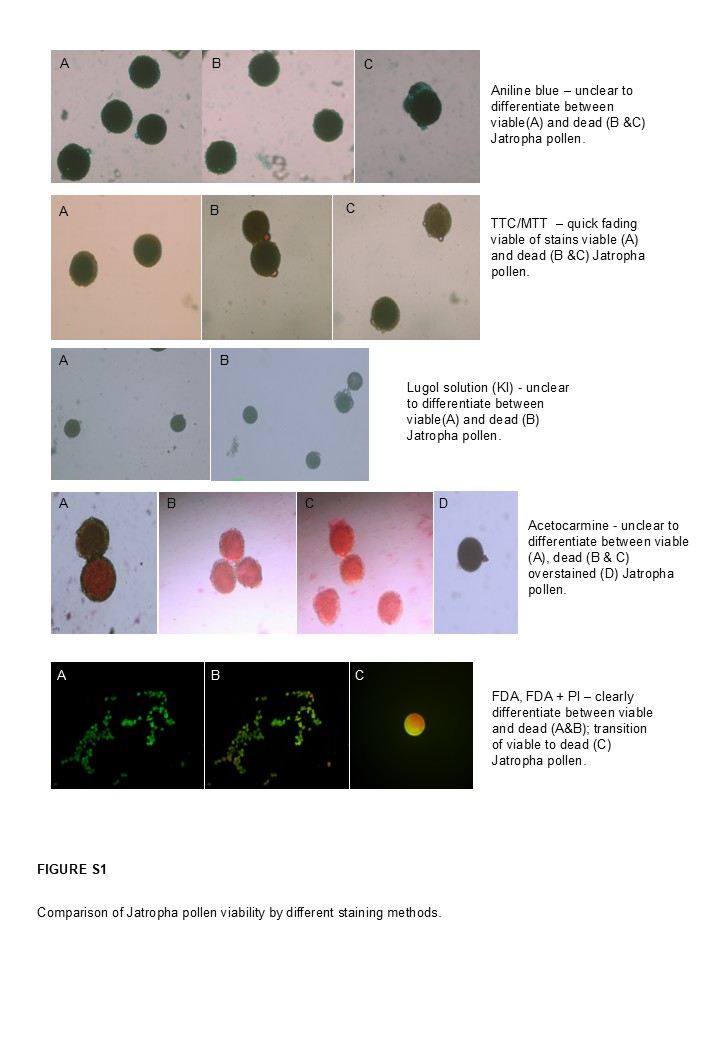

Supplement: Supplementary file 1 [file DataSheet1.docx]
